# Comparative Genome and Transcriptome Integration Studies Reveal the Mechanism of Pectoral Muscle Development and Function in Pigeons

**DOI:** 10.3389/fgene.2021.735795

**Published:** 2021-12-21

**Authors:** Haobin Hou, Xiaoliang Wang, Changsuo Yang, Xia Cai, Wenwei Lv, Yingying Tu, Aodungerile Bao, Quanli Wu, Weimin Zhao, Junfeng Yao, Weixing Ding

**Affiliations:** ^1^ Shanghai Academy of Agricultural Sciences, Shanghai, China; ^2^ National Poultry Engineer Research Center, Shanghai, China; ^3^ Shanghai Jinhuang Pigeon Company, Shanghai, China

**Keywords:** pigeons, pectoral muscle, selective sweep, transcriptome, candidate gene

## Abstract

Pigeon breed resources provide a genetic model for the study of phenomics. The pectoral muscles play a key role for the meat production performance of the meat pigeon and the athletic ability of the High flyers. Euro-pigeons and Silver King pigeons are commercial varieties that exhibit good meat production performance. In contrast to the domestication direction of meat pigeons, the traditional Chinese ornamental pigeon breed, High flyers, has a small and light body. Here, we investigate the molecular mechanism of the pectoral muscle development and function of pigeons using whole-genome and RNA sequencing data. The selective sweep analysis (*F*_*ST*_ and log2 (θπ ratio)) revealed 293 and 403 positive selection genes in Euro-pigeons and Silver King, respectively, of which 65 genes were shared. With the Silver King and Euro-pigeon as the control group, the High flyers were selected for 427 and 566 genes respectively. There were 673 differentially expressed genes in the breast muscle transcriptome between the commercial meat pigeons and ornamental pigeons. Pigeon genome selection signal combined with the breast muscle transcriptome revealed that six genes (*SLC16A10*, *S100B*, *SYNE1*, *HECW2*, *CASQ2* and *LOC110363470*) from commercial varieties of pigeons and five genes (*INSC*, *CALCB*, *ZBTB21*, *B2M* and *LOC110356506*) from Chinese traditional ornamental pigeons were positively selected which were involved in pathways related to muscle development and function. This study provides new insights into the selection of different directions and the genetic mechanism related to muscle development in pigeons.

## Introduction

Archaeological evidence indicates that pigeons were historically consumed as food for thousands of years (Blasco et al., 2014) and represented an important protein source for humans. In Europe, North America, and Asia, squab is considered a delicacy and is very popular among consumers (Kokoszyński et al., 2020). The pigeon industry gradually emerged in China during the early 1970s. After nearly 40 years of development, the number of pigeons in stock, out of stock, and the total production of pigeons ranked in China first in the world. According to the 2018 China Poultry Industry Development Report, there were 256,000 pairs of grandparent breeding pigeons, 41.2 million pairs of parent breeding pigeons, an increase of 5% over 2017, and an annual production of 643 million squabs (China Animal Agriculture Association, 2018). Similar to other types of livestock and poultry, breeders tend to cultivate large sized, full-breasted pigeons with high fecundity. There are substantial differences in body shape among different pigeon breeds. The largest pigeon can reach 1,000 g and small pigeons can reach to 250 g, which is nearly a four-fold difference in body mass (Stringham et al., 2012). For example, Euro-pigeon and Silver King weigh over 600 g at 4 weeks of age (Pomianowski et al., 2009), whereas some local varieties only weigh 250 g (Zhang et al., 2020). In contrast to meat pigeons, some performance and carrier pigeons are selected for athletic capability, and these pigeons are generally highly adept at flying. Carrier pigeons have the capacity for long-distance exploration and are known as messengers (Biro et al., 2007), whereas flipping pigeons are recognized as air dancers. High flying pigeons are more famous for their high altitude flying and also have important ornamental traits.

Most studies investigating quality traits using pigeon genomics have focused on the feather crown (Shapiro et al., 2013), feather color (Vickrey et al., 2018), and foot feathers (Domyan et al., 2016; Boer et al., 2019). Moreover, analyses of complex traits have mainly focused on competition ability (Shao et al., 2020). Using genomics and transcriptomics, researchers have revealed that the *CASK* gene is the key gene involved in the homing ability of carrier pigeons (Gazda et al., 2018). The relationship between candidate gene polymorphisms and meat quality traits has also been studied (Ye et al., 2016; Mao et al., 2018; Dong et al., 2019), and the physical and chemical characteristics of pigeon meat were previously reported (Kokoszyński et al., 2020). Many studies have found that the *MSTN* gene is related to muscle development in cattle (Berry et al., 2002; Miretti et al., 2013), pigs (Stinckens et al., 2008; Li et al., 2020), chickens (Guernec et al., 2004; Dushyanth et al., 2016), and sheep (Grochowska et al., 2019). In addition, *MSTN* gene expression in the breast muscle of pigeons was found to be significantly higher than that in other tissues, and to increase with age (Liu et al., 2019).

The genetic mechanism of body weight and size traits in pigs (Gusev et al., 2016), horses (Srikanth et al., 2019), and ducks (Wang L. et al., 2017) was revealed using multi-omics; however, few genome-wide studies have been conducted on gene mapping of pigeon growth traits, particularly the development of pectoral muscles. Pigeon breast muscle accounted for approximately 30% of the slaughter weight (Kokoszyński et al., 2020). Moreover, the growth and development of breast muscle is extremely important for meat performance and the flying ability of pigeons. In this study, we selected three breeds and resequenced a total of 23 individuals, including eight Euro-pigeons (EU), eight Silver King pigeons (SK), and seven High flyers. European meat pigeons and SK pigeons are larger and have better meat performance. HF are good at flying at high altitudes, with the characteristics of a small size and light posture. The distinct purposes of the pigeons diverged these breeds via human selection. The selection signal revealed genes that were positively selected among each of the breeds. Next, 28-day-old pigeon breast muscle transcriptome differences combined with the selection signal revealed the key genes required for pigeon muscle growth. This study is of great significance for the discovery of candidate genes that affect pigeon growth and development traits, and provides new insight into muscle development and function of altricial birds using functional omics research.

## Materials and Methods

### Pigeons and Ethics Approval

The whole genome of 23 pigeons, including eight European meat pigeons, eight SK pigeons, and seven HF pigeons were resequenced. All pigeons are from Shanghai Jinhuang Pigeon Industry Co., Ltd. After blood collection from the pterygoid vein, DNA was extracted using a Tiangen kit, and the DNA concentration was detected with a Nanodrop Spectrophotometer 2000. An Illumina Hiseq PE150 sequencing platform was used for sequencing. Pigeons used in this study were approved by the Ethics and Animal Welfare Committee of Shanghai Academy of Agricultural Sciences (No. SAASPZ0521012).

### Comparison of Reference Genomes

The effective high quality sequencing data were compared to the reference genome (reference genome download link: https://www.ncbi.nlm.nih.gov/genome/10719?genome_ assembly_ id = 39619) using BWA (Li and Durbin 2009) software (parameter: mem-t 4-K 32-m), and comparison results were removed with SAMTOOLS (Li et al., 2009) (parameter: rmdup).

### SNP Detection and Annotation

We used SAMTOOLS (Li et al., 2009) to detect population SNPs, and ANNOVAR (Wang et al., 2010) software was used for SNP annotation. The Bayesian model was used to detect polymorphic loci in the population, and quality control SNPs were obtained through the following filtering and screening method: 1) Q20 quality control (SNPs with a quality value of Q20 (i.e., the sequencing error rate was greater than 1% were filtered out); 2) the SNPs were at least five bp apart from each other (Since the probability of two SNPs being so close is extremely low, it is considered to be due to errors in sequencing, experimental factors, or analysis, and the two SNPs are removed); 3) the support number (coverage depth) of the SNP was between [1/3, five] times of the average depth.

### Analysis of Pigeon Population Structure

We used neighbor joining methods to construct the cluster tree. After SNP detection, the individual SNPs can be used to calculate the distance between populations. TreeBeST-1.9.2 software (https://mybiosoftware.com/treebest-1-9-2-softwares-phylogenetic-trees.html) was used to calculate the distance matrix. Using the distance matrix, a cluster tree was constructed using the neighbor joining method. The bootstrap values were calculated 1,000 times. We used EIGENSOFT (v5.0; https://www.hsph.harvard.edu/alkes-price/software/) for principal component analysis (PCA) on an individual scale for the 23 pigeons.

### Analysis of Selection Signal Based on FST and θπ

We calculated the genome-wide distribution of *F*_*ST*_ values (Weir and Cockerham 1984) and θπ ratios among the threepigeon breeds using a sliding-window approach (40-kb windows with 20-kb increments). The θπ ratios were log2(θπ ratio) transformed. We considered the windows with the top 5% values for the *F*_*ST*_ and log2(θπ ratio) simultaneously as candidate outliers under strong selective sweeps. Furthermore, the overlap information of a selected signal was obtained using the “vennDiagram” package in R (https://www.omicstudio.cn/tool/6).

### Comparative Transcriptome Analysis of the Pectoral Muscle

Twelve healthy 28-day-old female pigeons were used as research materials. The breeding method was caged and free-feeding. The breast muscles of four EU meat pigeons, four SK pigeons, and four HF pigeons were collected for transcriptome analysis, and the slaughter traits were recorded (Table 1). Total RNA was isolated and purified using TRIzol reagent (Invitrogen, Carlsbad, CA, United States) in accordance with the manufacturer’s procedure. The amount and purity of the RNA in each sample was quantified using a NanoDrop ND-1000 (NanoDrop, Wilmington, DE, United States). RNA integrity was assessed using a Bioanalyzer 2100 (Agilent, CA, United States) with an RIN number >7.0, and confirmed by electrophoresis with denaturing agarose gel. Finally, we performed 2 × 150 bp paired-end sequencing (PE150) with an Illumina Novaseq™ 6,000 (LC-Bio Technology Co., Ltd., Hangzhou, China) following the vendor’s recommended protocol.

**TABLE 1:** Slaughter traits of three pigeon breeds.

Breeds	Live weight (g)	Slaughter weight (g)	Eviscerated weight (g)	Pectorales weight (g)
Euro-pigeon	599.25 ± 64.20^a^	523.00 ± 65.56^a^	404.50 ± 50.49^a^	119.5 ± 19.21^a^
Silver King	482.75 ± 34.54^b^	423.50 ± 34.38^b^	331.50 ± 21.27^b^	86.00 ± 5.89^b^
High flyer	311.00 ± 32.76^c^	262.50 ± 32.80^c^	177.00 ± 25.53^c^	64.95 ± 9.16^c^

The data in the same column are superscripted with different lowercase letters (a, b, and c), indicating significant differences (*p* < 0.05).

### Sequence and Primary Analysis

Cutadapt software (https://cutadapt.readthedocs.io/en/stable/, version: cutadapt-1.9) was used to remove the reads that contained adaptor contamination (command line: ∼cutadapt -a ADAPT1 -A ADAPT2 -o out1. fastq -p out2. fastq in1. fastq in2. fastq -O 5 -m 100). After removing the low quality and undetermined bases, HISAT2 software (https://daehwankimlab.github.io/hisat2/, version: hisat2-2.0.4) was used to map reads to the Cliv_1.0 rock pigeon reference genome (command line: ∼hisat2 -1 R1. fastq.gz -2 R1. fastq.gz -S sample_mapped.sam). The mapped reads of each sample were assembled using StringTie (http://ccb.jhu.edu/software/stringtie/, version: stringtie-1.3.4d. Linux_x86_64) with default parameters (command line: ∼stringtie -p 4 -G genome. gtf -o output. gtf -l sample input. bam). The transcriptomes from all of the samples were merged to reconstruct a comprehensive transcriptome using GffCompare software (http://ccb.jhu.edu/software/stringtie/gffcompare.shtml, version: gffcompare-0.9.8. Linux_x86_64). After the final transcriptome was generated, StringTie and Ballgown (http://www.bioconductor.org/packages/release/bioc/html/ballgown.html) were used to estimate the levels of transcript expression and determine the mRNA expression level by calculating FPKM (FPKM = [total_exon_fragments/mapped_reads (millions) × exon_length (kB)]) (command line: ∼stringtie -e -B -p 4 -G merged. gtf -o samples. gtf samples. bam). The differentially expressed mRNAs with a fold-change > 2 or fold-change < 0.5, and p-value < 0.05 were selected using DESeq2(Pertea et al., 2015) (http://www.bioconductor.org/packages/release/bioc/html/DESeq2.html).

### Gene Ontology Enrichment and Pathway Analysis

The function of the differentially expressed genes in different types of pigeon breast muscle was investigated by comparing the following groups: EU vs SK, EU vs HF, and SK vs HF. Goseq was used to perform the enrichment analysis of the GO term (Young et al., 2010). Based on the KEGG database, KOBAS 2.0 software was used to analyze the pathways associated with the differentially expressed genes (Kanehisa et al., 2008). Ggplot two was used to analyze the enrichment of the GO and KEGG databases, and the results were displayed as a scatter plot (bubble chart).

### Real-Time Quantitative PCR Analysis

Using 12 RNA samples isolated from pigeons (four EU pigeons, four SK pigeons, and four HF pigeons), the RNA OD value was detected with an ultra-micro nucleic acid protein analyzer (scandrop100). SYBR Green I was used to detect the expression of target genes in the samples using the A260/A280 ratio, β-actin as an internal reference gene, and four target genes. The fluorescence quantitative PCR program and system was performed as follows: Step 1: 95°C for 3 min; Step 2: 95°C for 10 s; Step 3: 60°C for 30 s + plate read; repeat step 2 for 39 cycles. Melt curve analysis: (60°C–95 °C, +1°C/cycle, holding time of 4 s).

## Results

A total of 23 samples were sequenced from different pigeons, with high quality data volume of 199.3 Gb, high sequencing quality (Q20 ≥ 96.0%, Q30 ≥ 90.2%), and normal GC distribution. None of the 23 samples were contaminated (Supplementary Table S1). The library was successfully constructed and sequenced. The average map rate of the population samples was 97.6%, the average sequencing depth of the genome (excluding gap regions) was 7.30 (only reads with a comparison quality >0 are considered), and the average coverage was 98.8% (at least one base is covered) (Supplementary Table S2). A total of 5,673,290 SNPs were detected (Supplementary Table S3).

### Population Structure

**FIGURE 1:**
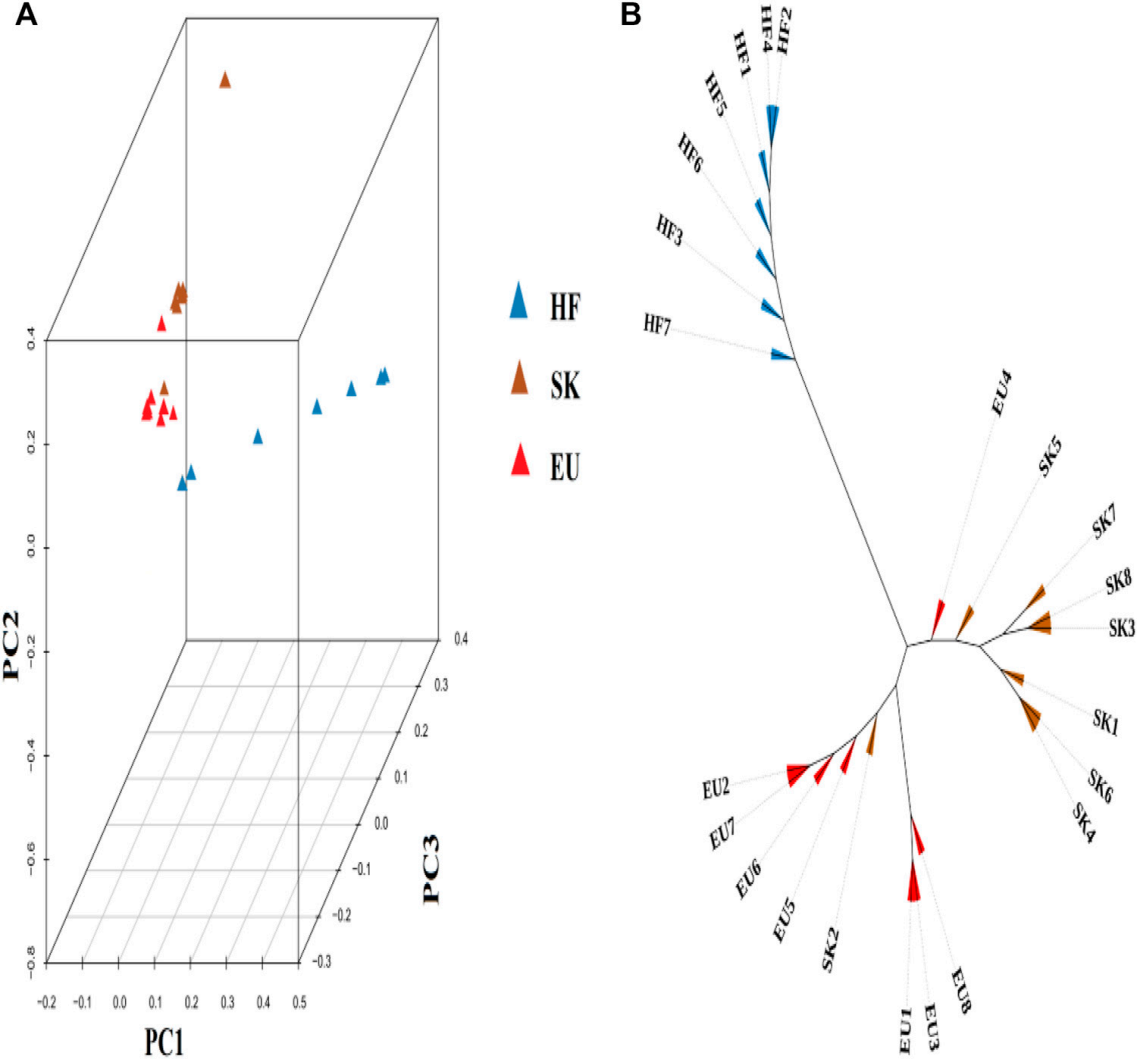
**(A)** The 3D PCA plot of pigeon population. EU (Euro-pigeon), SK (Silver King), HF (High flyers). **(B)** Cluster tree of three pigeon breeds.

Based on the degree of SNP differences among individuals of different pigeon breeds, PCA analysis revealed that EU and SK had a closer genetic distance, while Chinese traditional ornamental pigeon and commercial meat pigeon had a longer genetic distance (Figure 1A). The cluster tree analysis revealed that commercial meat pigeon breeds and HF pigeons formed two independent branches, indicating that there was a large genetic distance between them. Although there are obvious differences in feather color between EU pigeons and SK pigeons, the genetic distance between them was relatively close (Figure 1B).

### Positive Selection of Meat Pigeon and High Flyers

**FIGURE 2:**
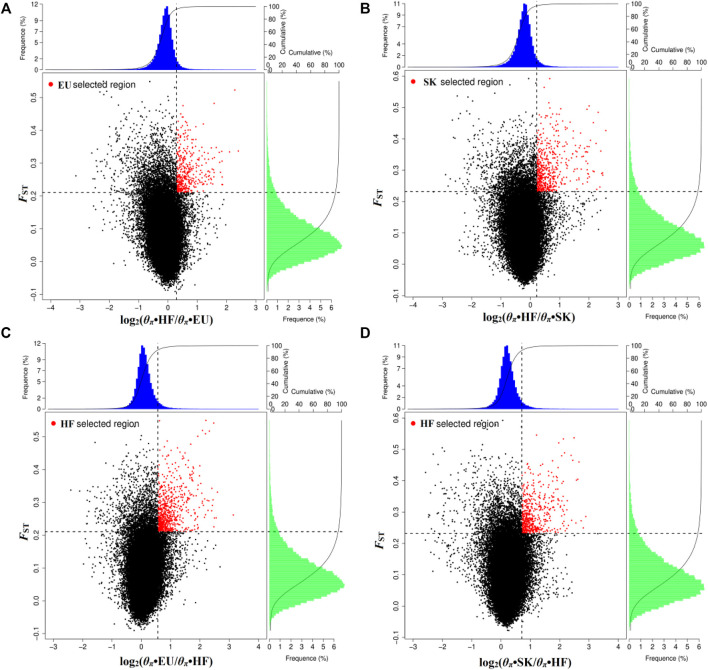
Distribution of log_2_ values (*θ*_*π*_•control/*θ*_*π*_•selected) and the top 5% highest *F*_ST_ values calculated in 40-kb sliding windows with 20-kb increments. **(A)** Compared with HF, EU are subject to positive selection of genomic regions. **(B)** Compared with HF, SK are subject to positive selection of genomic regions. **(C)** Compared with EU, HF are subject to positive selection of genomic regions. **(D)** Compared with SK, HF are subject to positive selection of genomic regions.

By analyzing selection signals, 293 genes were positively selected in the EU population and 403 genes were positively selected in the SK population. There were 65 overlapping genes (Supplementary Table S4; Figures 2A,B). When EU was used as a control group, 566 genes were selected by HF pigeons. When SK was used as the control group, 427 genes were selected by HF pigeons. There were 262 overlapping genes (Supplementary Table S4; Figures 2C,D).

### Comparative Transcriptome of the Pectoral Muscle

A total of 12 breast muscle samples (4 EUs, 4 SKs and 4 HFs) of three pigeon breeds were sequenced according to the standard operation, and a total of 94.73 G of raw data was obtained. After performing quality control, 92.48 G of clean data were obtained, 97.62% of which were effective reads. The proportion of bases with quality values ≥20 (sequencing error rate less than 0.01) was 99.97%, the proportion of bases with quality values ≥30 (sequencing error rate less than 0.001) was 98.11%, and the proportion of GC content was 48.42% (Supplementary Table S5). Hisat was used to compare the reference genome of valid data after preprocessing, revealing a comparison rate of 90.86% (Supplementary Table S6). The percentage of exon annotated sequences was 91.63%, whereas the percentage of intron and intergenic reads was 4.78 and 3.59%, respectively (Supplementary Figure S1).

### Analysis of Differentially Expressed Genes (DEGs)

**FIGURE 3:**
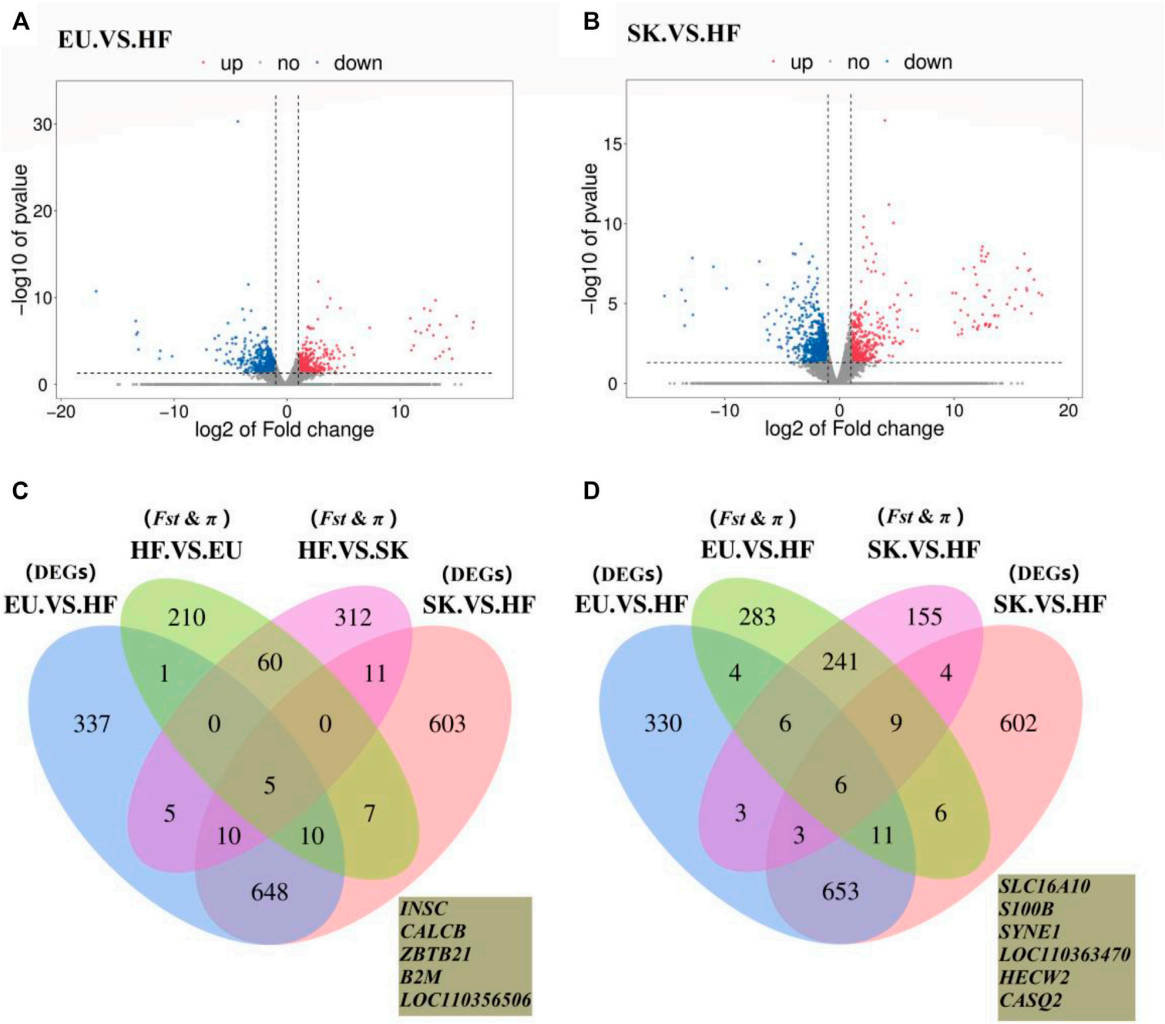
**(A)** Volcano plot of EU. VS. HF breast muscle differential gene expression level. **(B)** Volcano plot of SK. VS. HF breast muscle differential gene expression level. **(C)** Venn diagram of the commercial pigeon positive selection signal combined with DEGs analysis of the breast muscle transcriptome. **(D)** Venn diagram of the HF positive selection signal combined with DEGs analysis of the breast muscle transcriptome.

The expression profiles of 26,640 genes were obtained using Ballgown package to provide file input for fpkm quantification. The genes with FC > 2 times or FC < 0.5 times and *p* value <0.05 were defined as differentially expressed genes. A total of 1,016 differentially expressed genes were obtained by comparing the transcriptome of breast muscle samples from EU and HF pigeons, including 408 up-regulated genes and 608 down-regulated genes (Supplementary Table S7; Figure 3A). There were 1,294 differentially expressed genes between SK and HF pigeons, including 465 up-regulated genes and 829 down-regulated genes (Supplementary Table S8; Figure 3B). A total of 322 genes were identified in EU vs SK group, of which 195 were up-regulated and 127 were down regulated (Supplementary Table S9).

The results showed that there were more differences in breast muscle gene expression between meat pigeons and HF; however, there were fewer differences between meat pigeons, which was consistent with the phenotypic analysis results. Further analysis with a Wayne diagram showed that there were 673 overlapping genes in the EU *vs*. HF and SK vs HF groups (Figure 3). Pigeon genome selection signal combined with the breast muscle transcriptome revealed that six genes from commercial varieties of pigeons (Figure 3C) and five genes from Chinese traditional ornamental pigeons (Figure 3D) were positively selected.

### GO and KEGG Enrichment Analysis

**FIGURE 4:**
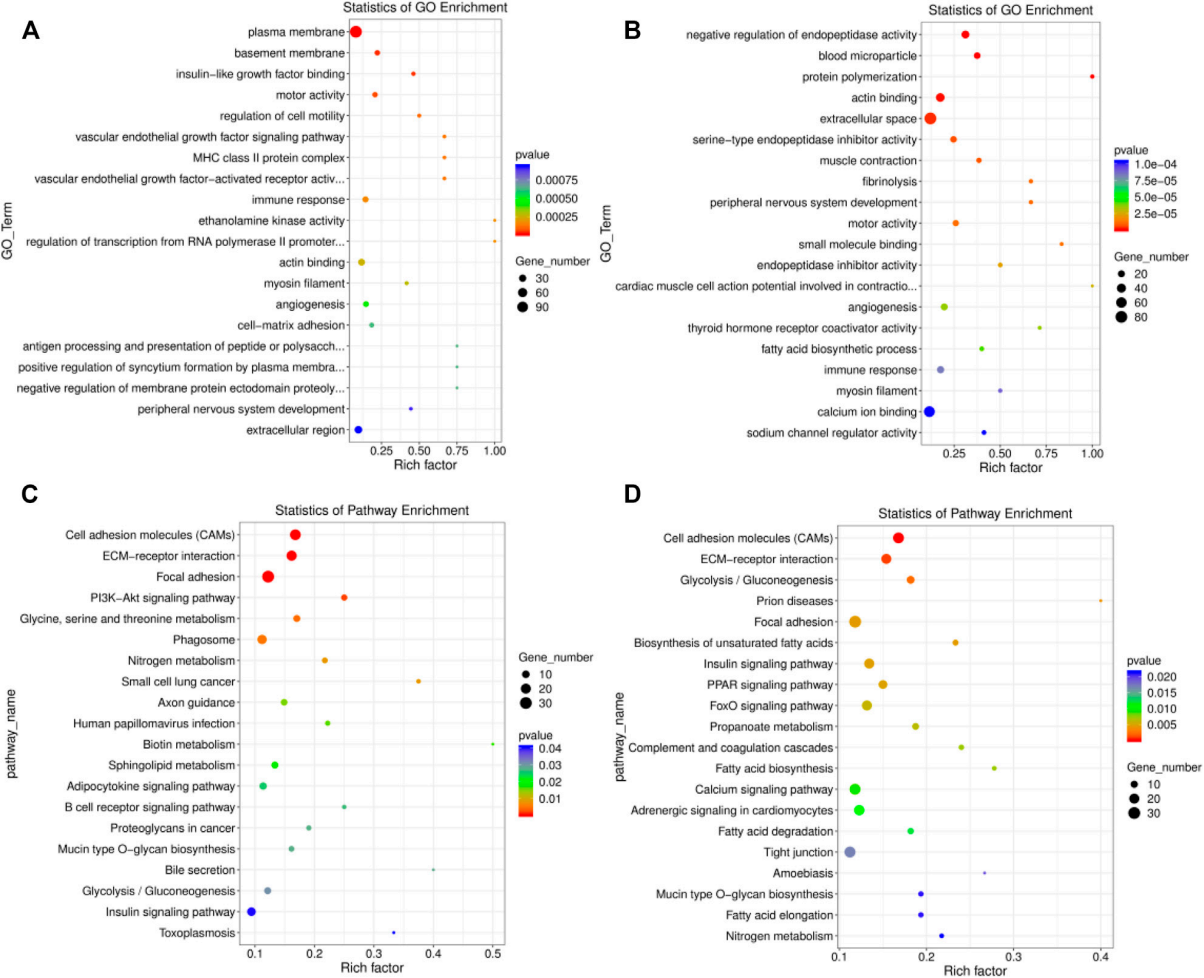
Top 20 significantly enriched GO and KEGG pathways of the differentially expressed genes. **(A)** The top 20 significant GO terms in the EU *vs*. HF group. **(B)** The top 20 significant GO terms in the SK *vs*. HF group. **(C)** The top 20 significant KEGG pathway in the EU *vs*. HF group. **(D)** The top 20 significant KEGG pathway in the SK vs HF group.

A total of 305 significant GO terms were identified in the EU *vs*. HF group, the top 20 of which were related to muscle function, including actin binding (GO:0003779, *p* = 0.0003) and myosin filament (GO:0032982, *p* = 0.0003) (Supplementary Table S10; Figure 4A). Moreover, the insulin-like growth factor binding (GO:0005520, *p* = 0.00003) pathway, which is related to growth and development, was also enriched. For SK and HF, the differentially expressed genes were enriched in 358 significant pathways (Supplementary Table S11; Figure 4B). Similarly, the first 20 pathways included actin binding (GO:0003779, *p* = 0.000) and myosin filament (GO:0032982, *p* = 0.00009). In addition, muscle contraction (GO:0006936) was also related to muscle function. Although 516 GO terms were enriched in the EU *vs*. SK, the top 20 GO terms were not related to muscle development or function (Supplementary Table S12).

A KEGG pathway enrichment analysis of the differentially expressed genes in the breast muscle tissue of commercial meat pigeons and HF was carried out. The differentially expressed genes in the breast muscle of EU and HF were significantly enriched in 217 pathways, 21 of which were extremely significantly enriched (*p* < 0.05) (Supplementary Table S13; Figure 4C). The differentially expressed genes in the breast muscle of SK and HF were significantly enriched in 178 pathways, of which 31 were extremely significantly enriched (*p* < 0.05) (Supplementary Table S14; Figure 4D). The significantly enriched pathways exhibited by the two meat breeds included participation in cell proliferation, differentiation, metabolism, and synthesis. These pathways included cell adhesion molecules (CAMs), ECM-receptor interaction, glycolysis/gluconeogenesis, focal adhesion, insulin signaling pathway, mucin type O-glycan biosynthesis, biotin metabolism, and the adipocytokine signaling pathway. Differential gene expression in the breast muscle of meat pigeon breeds was enriched in 111 pathways, 19 of which were significantly enriched (*p* < 0.05) (Supplementary Table S15).

### Verification of Key Gene Expression

**FIGURE 5:**
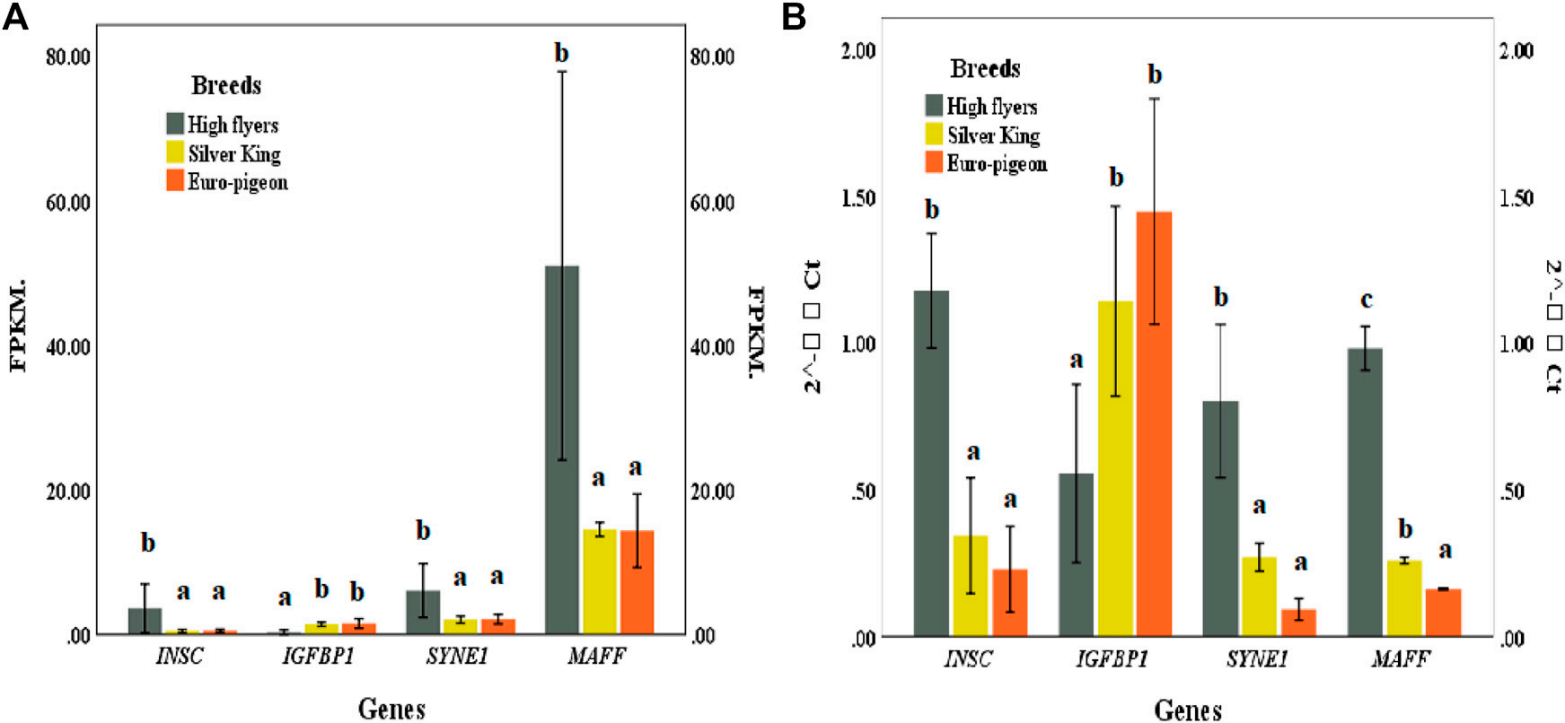
**(A)** Gene expression of pigeon breast muscle transcriptome sequencing. FPKM (Fragments Per Kilobase of exon model per Million mapped reads). **(B)** Expression of five significant differentially expressed genes validated by qRT-PCR. The same lowercase letters in the histogram represent no significant difference (*p* > 0.05), and different letters represent significant difference (*p* < 0.05).

Four genes, including *SYNE1*, *INSC*, *IGFBP1* and *MAFF* exhibited significant different expression patterns between the meat breeds and the HF breed. The expression patterns of these genes in pigeon breast muscle were consistent with the RNA sequencing results, which verified the accuracy of the comparative transcriptome sequencing results (Figure 5; Supplementary Table S16).

## Discussion

In this study, a genomics perspective was used to analyze the genes of two meat pigeon breeds (EU and SK) and an ornamental breed. To further study the genetic mechanism associated with the phenotype differences in the pectoral muscle between the two different breeds, a comparative transcriptome was used to reveal the key genes related to growth and development and more importantly, to discover genes that regulate muscle function. The selection signal combined with the comparative transcriptome revealed *INSC*, *CALCB*, *ZBTB21*, *B2M*, and *LOC110356506* as the genes that affect breast muscle development in meat pigeons. *INSC* can regulate mesoderm differentiation of mouse embryonic stem (ES) cells (Ishibashi et al., 2016). In addition, the human *INSC* gene, which is closely associated with the *CALCB* gene by an interval of about 30 kb, was assigned to human chromosome 11p15.2-p15.1 (Katoh and Katoh 2003). While a short-term RNA interference-mediated *CALCB* knockdown had no effect on the proliferation and clonogenic growth of ES cells *in vitro*, its long-term knockdown decreased ES growth both *in vitro* and *in vivo*. The *INSC* gene also plays an important role in the growth and development of meat pigeon breast muscle. In the differential expression analysis, 673 genes were identified, among which the genes related to growth and development included *IGFBP1*, *IGFBP4*, *FOXO3*, *HMGA1*, and *FAM184B*. Studies have shown that the *IGFBP1* gene has an important regulatory effect on fetal growth and development. Both hypoxia and leucine deprivation can increase the level of the *IGFBP1* gene expression and phosphorylation, inhibit the effect of *IGF*, and lead to impaired embryonic development (Kajimura et al., 2005; Seferovic et al., 2009). *IGF-1* and *IGFBP1* have anabolic effects on skeletal muscle and are related to the preservation of lean meat (Velloso 2008). Consequently, *IGFBP1* play a role in the growth and metabolism of pigeons and regulate the development of the pectoral muscle.

Compared with commercial meat pigeons, HFs are selected for light weight individuals. At the same time, the flying ability of HFs is inferior to that of carrier pigeons, characterized by a short hovering distance and slow flying speed. Genomic and transcriptomic analyses revealed that *SLC16A10*, *S100B*, *SYNE1*, *HECW2*, and *CASQ2* genes were positively selected. Studies in mice have shown that the *SLC16A10* gene is involved in promoting the cellular transport of thyroid hormone (Müller et al., 2014). Studies in pigs have demonstrated that a low protein diet induces higher expression of the *SLC16A10* gene, resulting in limited protein synthesis and growth of the longissimus dorsi (Wang D. et al., 2017). Mutations in the *HECW2* gene can cause neurodevelopmental delay, and the clinical features shared by patients include severe developmental delay and hypotonia (Berko et al., 2017). In addition, this gene mutation has also been linked to epilepsy-associated developmental delay (Ullman et al., 2018). *CASQ2* plays an important role in regulating Ca^2+^ release in the sarcoplasmic reticulum, buffering of Ca^2+^ in the sarcoplasmic reticulum, and promoting the closure of cardiac ryanodine receptors during diastole (Györke and Terentyev 2008). *CASQ2* is expressed in slow muscle throughout the lifespan of mice, but only in fast muscle during the newborn stage and early development. Indeed, *CASQ2* ^−/−^ mice display ultrastructural changes only in the rapid twitch muscle (Valle et al., 2016).

A defect in nesprin-1 encoded by the *SYNE1* gene can cause Emery-Dreifuss muscular dystrophy (EDMD), which is characterized by joint contracture, myasthenia, and cardiac abnormalities (Zhang et al., 2007; Pillers and Von Bergen 2016; Chen et al., 2017; Heller et al., 2020). The protein encoded by *SYNE1* is widely expressed in a variety of tissues and connects the outer membrane of the nuclear membrane with the cytoskeleton by interacting with F-actin (Rajgor and Shanahan 2013) and is highly expressed in striated muscle (Zhang et al., 2010). At the same time, several studies throughout the world have reported that *SYNE1* gene mutations can cause autosomal receptive cerebellar ataxia type 8 (SCAR8) (Izumi et al., 2013; Noreau et al., 2013; Hamza et al., 2015; Mademan et al., 2016; Synofzik et al., 2016). The *SYNE1* gene was selected in HF pigeons and found to be significantly up-regulated (*p* < 0.05) compared with that in meat pigeons. The body weight and breast muscle weight of HF pigeons are significantly lower (*p* < 0.05) than those of commercial meat pigeons (Table 1), indicating that this gene has a negative regulatory effect on the growth and development of pigeons. With the enrichment analysis, *SYNE1* was found to be involved in actin binding (GO: 0003779) and muscle cell differentiation (GO: 0042692). The HF breed has a lighter body that is suitable for gliding in the air, but has a slower flight speed and is not suitable for long-distance flight. It is speculated that mutations in the *SYNE1* gene will affect the development and function of HF breast muscle.

## Conclusion

This study revealed the selection of genome regions between commercial meat pigeons and Chinese traditional ornamental pigeons using genomics analyses. The results of transcriptome sequencing and differential expression analysis revealed the molecular mechanism of breast muscle phenotypic differences between commercial meat pigeons and Chinese traditional ornamental pigeons. Multi-omics further revealed multiple genes related to cell differentiation, muscle development, and skeletal muscle function. Among these genes, *INSC* and *CALCB* were related to cell differentiation and were positively selected and up-regulated in both EU pigeons and SK pigeons. Thus, these genes may be involved in promoting the growth and development of the pigeon breast muscle. More importantly, we found that the *SYNE1* gene was related to actin binding (GO: 0003779) and muscle cell differentiation (GO: 0042692) in the ornamental pigeon population. This study provides a few genes that require further study of their molecular mechanisms related to muscle development, which will help understand the genomic imprints of different types of pigeons due to artificial selection.

## Data Availability

The datasets presented in this study can be found in online repositories. The names of the repository/repositories and accession number(s) can be found in the article/Supplementary Material.
