# Supplementary figures and images for: Comparative Genome and Transcriptome Integration Studies Reveal the Mechanism of Pectoral Muscle Development and Function in Pigeons
Source: Front Genet. 2021 Dec 21;12:735795. doi: 10.3389/fgene.2021.735795 (PMC8721168; doi:10.3389/fgene.2021.735795)

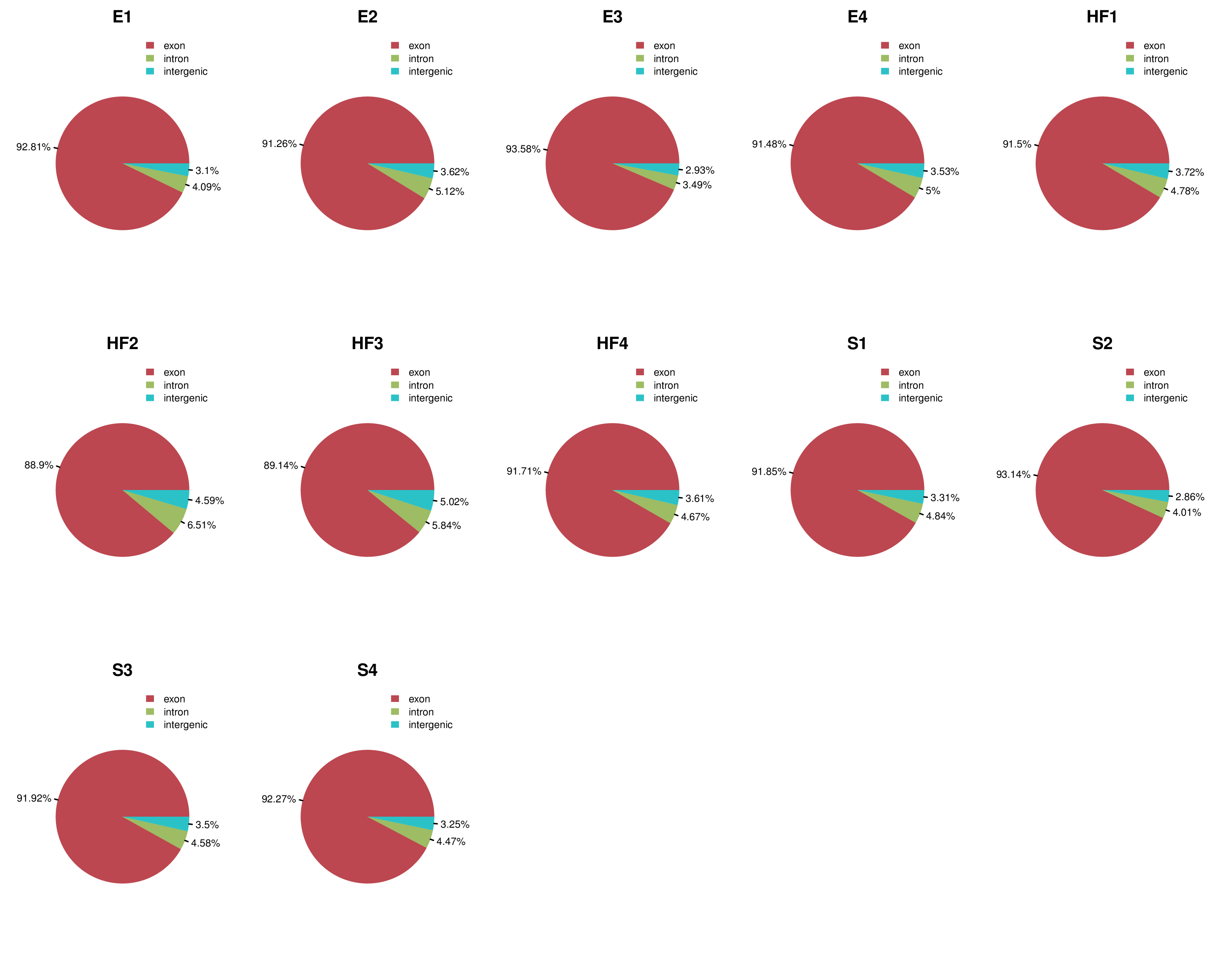

Supplement: Supplementary file 1 [file DataSheet1.ZIP › Supplementary material/Figure S1 Regional distribution statistics of reference genome mapped..png]
